# Platelet effector functions in inflammation

**DOI:** 10.1016/j.rpth.2026.103412

**Published:** 2026-03-17

**Authors:** Raphael Escaig, Lennart Kreutz, Rainer Kaiser, Leo Nicolai

**Affiliations:** 1 Medizinische Klinik und Poliklinik I, University Hospital Ludwig-Maximilian University, Munich, Germany; 2 German Centre for Cardiovascular Research (DZHK), partner site Munich Heart Alliance, Munich, Germany

**Keywords:** immunothrombosis, platelets, procoagulant, thromboinflammation, thrombosis

## Abstract

A State of the Art lecture titled “Platelet effector functions in inflammation” was presented at the International Society on Thrombosis and Haemostasis congress in 2025. It is increasingly recognized that platelets are critical orchestrators of innate and adaptive immune responses. In this review, we highlighted how these cells partially engage distinct receptors, signaling pathways, and effector functions to fulfill their role in immunity. In the inflamed microenvironment, platelets migrate along substrate gradients to optimize positioning to sites of injury or pathogen entry in an actin-related protein 2/3 complex-dependent manner. This allows for pathogen collection and bundling, as well as prevention of inflammatory bleeding. For the latter task, platelets integrate activation signals via glycoprotein VI and integrin αIIbβ3 to undergo procoagulant transformation, triggering local and restricted coagulation. We provided an overview of how these mechanisms are also associated with hyperinflammation and immunopathology, and how procoagulant activity is a driver of arterial and venous thrombosis. Moreover, we discussed that platelets increase their propensity for immune engagement over their lifespan while losing hemostatic prowess. Finally, we summarized additional relevant new data on this topic presented during the 2025 International Society on Thrombosis and Haemostasis Congress.

## 1 Introduction

Platelets have long been defined by their role in clot formation, yet a growing body of evidence positions them as “guardians of the vasculature” that continuously patrol the circulation, sense breaches in endothelial integrity, and shift between hemostatic and immune effector programs. In addition to sealing vessel injuries, platelets surveil inflamed microvessels and actively preserve barrier function by repositioning to microlesions, limiting inflammatory hemorrhage, and constraining pathogen dissemination [1,2]. This sentinel function has emerged within the broader framework of immunothrombosis, in which platelet-leukocyte-coagulation cross-talk serves host defense but, when dysregulated, drives thromboinflammation [[3], [4], [5]].

Platelet surveillance and rapid response depend on a tuned adhesion/signaling system. In clot formation, the canonical axes of glycoprotein (GP) Ib-IX-V/von Willebrand factor (VWF), GPVI/collagen, and integrin αIIbβ3/fibrin(ogen) cooperate to mediate initial capture, early activation, and subsequent stable attachment and aggregation, while driving cytoskeletal remodeling and secretion that amplify local signals [6,7]. The GPIb-IX-V complex mediates high-on/fast-off binding under shear through the VWF-A1 domain, swiftly inducing calcium mobilization and integrin activation. GPVI’s Fc receptor common γ chain-associated tyrosine kinase cascade, spleen tyrosine kinase (Syk)/phosphatidylinositol-specific phospholipase C γ 2 (PLCγ2), integrates signals from extracellular matrix components such as collagen engagement to amplify platelet activation and promote αIIbβ3 inside-out signaling via receptor conformational changes. The subsequent enhancement of αIIbβ3-mediated outside-in signaling through integrin engagement further drives platelet aggregation and stabilizes the developing hemostatic plug [6,7].

Importantly, these classical “hemostatic” receptors can also interact with inflammatory pathways and leukocyte ligands: GPIbα and platelet P-selectin can engage leukocyte Mac-1 (αMβ2) and P-selectin glycoprotein ligand-1 (PSGL-1), respectively, promoting platelet-leukocyte networking at inflamed endothelium and within developing thrombi [6,[8], [9], [10]]. This overlapping molecular interplay establishes a direct mechanistic link between the adhesion machinery and immune signaling modules (eg, toll-like receptors [TLRs] and immunoreceptor tyrosine-based activation pathways) highlighted in recent platelet signaling reviews [1].

The emerging view positions platelets as vascular sentinels equipped with a capture-and-signal receptor apparatus (eg, GPIb-IX-V/GPVI/αIIbβ3) that integrates with immune pathways, supported by cytoskeletal changes capable of context-specific effector responses in inflammation. This perspective redefines platelets not as passive clotting elements but as dynamic coordinators of vascular integrity and host defense. The remainder of this review builds this framework to examine platelet signaling modules and effector mechanisms that may be therapeutically leveraged to maintain barrier protection while mitigating thromboinflammatory risk.

## 2 Platelets in Clot Formation vs The Inflamed Microenvironment

**Figure 1:**
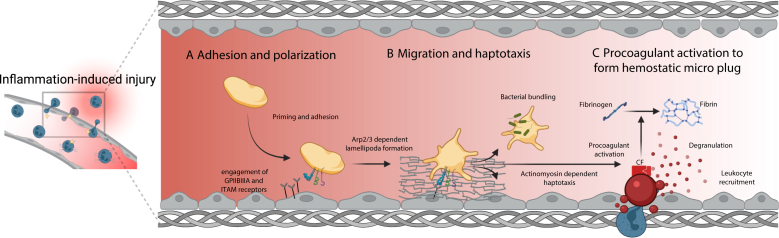
Platelet sentinels in inflamed microvessels **(**A) At sites of inflammation-induced endothelial injury, single platelets are captured under flow (via VWF/GPIb and GPVI-collagen), engage extracellular matrix components (eg, increased inside-out activation of αIIbβ3 and ITAM receptors), and polarize by forming Arp2/3 complex-dependent lamellipodia. (B) Polarized platelets then migrate along fibrinogen-decorated endothelial surfaces (haptotaxis) by coupling lamellipodial protrusion to actomyosin-dependent traction, allowing precise repositioning to microlesions and bundling of substrate-bound bacteria (mechano-scavenging). (C) Upon encountering matrix exposure at the breach sites, platelets switch to a procoagulant state with phosphatidylserine externalization that recruits plasmatic coagulation factors to the platelet surface and drives local fibrinogen to fibrin conversion. The result is a nonocclusive haemostatic micro-plug, which seals microdefects and preserves barrier integrity in inflammation (created by biorender.com). *Source:* Kaiser et al. 2023 [14]. Arp2/3, actin-related protein 2/3 complex; GP, glycoprotein; ITAM, immunoreceptor tyrosine-based activation motif; VWF, von Willebrand factor.

At sites of vascular injury, platelets engage in a conserved sequence of clot formation, with each step modulated by the biochemical and biophysical context of the vessel wall and local hemodynamics. It begins with receptor-mediated adhesion, advances through signal amplification and integrin activation, transitions to actomyosin-driven retraction, and results in thrombus propagation and stabilization. Work across intravital and in vitro platforms demonstrates that platelets dynamically adapt their morphology (lamellipodia-forming states), motility (ranging from stationary retraction to autonomous migration), and signaling profiles in response to the local matrix composition, shear microgradients, and inflammatory cues [2,[11], [12], [13]]. In inflamed microvessels, vascular fibrin(ogen) depositions “tag” microlesions and impose spatial gradients that bias platelet shape and migration (Figure 1 [14]). Conversely, in acute thrombosis, platelets are rapidly immobilized within 3-dimensional (3D) fibrin scaffolds, favoring contractility over migration [2,15,16].

The thrombus microenvironment induces coexisting platelet effector programs that support either growth/propagation or remodeling/consolidation. Genetic disruption of the nonmuscle myosin IIA heavy chain (MYH9) in megakaryocytes results in platelets that aggregate and secrete largely normally in suspension yet exhibit profoundly prolonged bleeding, absent clot retraction, disorganized thrombi, and increased embolization in vivo [17]. At the single-cell level, platelets behave as active contractile units that stiffen clots, aligning with the concept that the platelet actomyosin system is critical in reorganizing the fibrin network [12]. Mechanistically, endogenous fibrinolysis facilitates fibrin network flexibility and promotes efficient retraction in vivo, indicating that retraction and (limited) fibrinolysis are not antagonistic but synergistic processes in clot maturation [18]. These findings establish actomyosin contractility as a central determinant of thrombus consolidation and stable hemostasis, effectively decoupling thrombus architecture from primary platelet aggregation. Notably, the MYH9 model primarily reveals defects in thrombus growth and stability under conditions where platelets are densely packed within 3D fibrin scaffolds (eg, FeCl_3_-induced arterial injury), consistent with a role for the actomyosin system as a “clot consolidation” module in mechanically constrained thrombotic environments [17].

In contrast, the actin-related protein 2/3 c (Arp2/3) complex branched actin network underlies lamellipodia formation and platelet motility with little impact on classical clot retraction. Platelet-specific deletion of Arp2/3 complex subunit 2 (Arpc2) abrogates lamellipodia formation and haptotactic migration along fibrin(ogen) gradients but leaves tail bleeding and acute arterial thrombosis largely intact, thereby functionally separating “immune migratory” from “hemostatic” modules [2]. In models of lipopolysaccharide (LPS)-induced acute lung injury (ALI), C-C motif chemokine ligand 2-driven cremaster inflammation and peritonitis, and Arpc2-deficient platelets fail to reposition to neutrophil transmigration sites, which results in defective sealing of endothelial microlesions [2,19]. At the level of nucleation-promoting factors, loss of the cytoplasmic FMR1-interacting protein (Cyfip1)/Wiskott-Aldrich syndrome protein family verprolin-homologous (WAVE) complex eliminates large circular lamellipodia on rigid substrates but leaves residual Arp2/3 complex activity sufficient for limited motility. Accordingly, platelet factor 4 (PF4)CreCyfip1fl/fl mice show preserved vascular integrity in skin and lung inflammation despite defective lamellipodium formation [20]. Taken together, these studies indicate that the Arp2/3 complex-driven migratory competence becomes critical in inflamed microenvironments with extensive fibrin(ogen) tagging and transmigration-induced breaches, whereas it is dispensable for canonical thrombus formation.

Integrating these data suggests distinct functional hubs that are differentially engaged by thrombotic vs inflamed microenvironments. Myh9-dependent actomyosin contractility favors compaction, retraction, and thrombus stabilization within densely packed, 3D fibrin scaffolds [17], whereas Arp2/3 complex WAVE signaling equips platelets with lamellipodia for exploratory motility and haptotactic repositioning along endothelial fibrinogen deposits in inflamed venules, thereby enabling surveillance and sealing of inflammatory microlesions [2,19,20].

## 3 Platelet-Neutrophil Interplay in Inflammation

**Figure 2:**
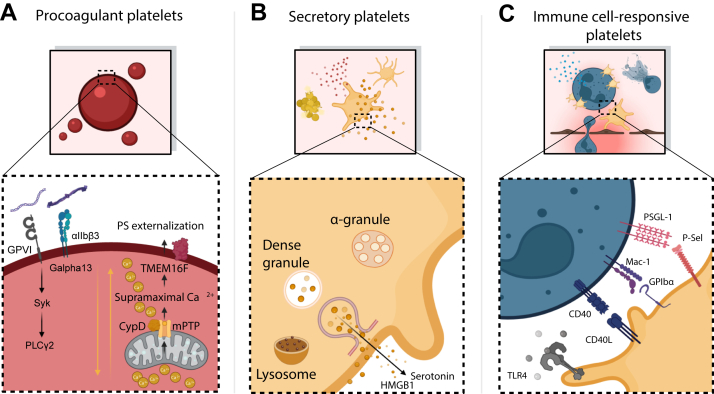
Platelet effector functions: triggers and signaling hubs Platelet responses in inflammation can be grouped into 3 frequently overlapping effector functions that are initiated by defined extracellular triggers and characteristic intracellular signaling hubs. (A) Procoagulant platelets. Costimulation via collagen—GPVI and integrin αIIbβ3 (GPIIb/IIIa)—Gα13 drives sustained cytosolic Ca^2+^ level rises that open the cyclophilin-D-dependent mitochondrial permeability transition pore (CypD-mPTP) and activate the Ca^2+^-dependent scramblase TMEM16F (*ANO6*), resulting in phosphatidylserine (PS) externalization and the ballooned procoagulant phenotype. These changes create a catalytic surface for thrombin generation and fibrinogen accrual at sites of endothelial breach. (B) Secretory platelets. Engagement of hemostatic receptors induces exocytosis from α-granules (eg, P-selectin translocation, HMGB1 release) and dense granules (eg, serotonin/5-HT), as well as lysosomal secretion, amplifying local platelet activation and tuning immune and endothelial responses. (C) Immune cell-responsive platelets. Platelets form an interface with leukocytes through selectin and integrin receptor engagement (P-selectin-PSGL-1; GPIbα-Mac-1) and costimulatory receptor pairs (CD40-CD40L) and sense danger signals via pattern-recognition receptors (eg, TLR4). These pathways organize platelet-leukocyte aggregates and prime neutrophil effector functions such as NETosis, increased transmigration, and degranulation within inflamed microvessels (created by biorender.com). *Source:* Kaiser et al. [23] 2025. 5-HT, 5-hydroxytryptamine; *ANO6*, *anoctamin 6*; CD40L, CD40 ligand; GP, glycoprotein; HMGB1, high mobility group box 1; NET, neutrophil extracellular trap; PSGL, P-selectin glycoprotein ligand-1; TLR4, toll-like receptor; TMEM16F, transmembrane protein 16F.

Platelets act as intravascular sentinels that license neutrophil pathogenicity within minutes of damage induction. In ALI and in systemic inflammatory models, platelets rapidly expose P-selectin, bind neutrophil PSGL-1 to form platelet-neutrophil aggregates (PNAs) in the circulation and at injury sites [21,22] (Figure 2 [23]).

PNAs are not byproducts of inflammation but upstream effectors that shape disease trajectories. In acid-induced lung injury and sepsis, platelet P-selectin-dependent PNAs amplify neutrophil adhesion in the pulmonary microvasculature, trigger thromboxane A_2_ generation, up-regulate endothelial intercellular adhesion molecule 1, and thereby escalate permeability and hypoxemia. Conversely, platelet depletion, therapeutic P-selectin blockade after injury, or thromboxane receptor antagonism each improve gas exchange, reduce neutrophil recruitment, normalize permeability, and prolong survival [22]. At the single-cell level, intravital imaging explains how these aggregates are generated and become pathogenic: once arrested in inflamed venules, neutrophils extend a PSGL-1-enriched uropod that “scans” the lumen for activated (P-selectin^+^) platelets. PSGL-1 engagement induces outside-in signals that reorganize Mac-1 (CD11b/CD18) and C-X-C chemokine receptor 2 across the neutrophil surface, optimizing intraluminal crawling and repositioning at endothelial junctions for transmigration [10]. The leukocyte integrin Mac-1 can directly ligate platelet GPIbα to stabilize platelet-neutrophil interactions and promote thrombosis with a favorable bleeding profile when selectively inhibited [24].

Together, these mechanistic and interventional data identify the platelet P-selectin-PSGL-1 axis as the initiating liaison that couples platelet activation to neutrophil behavioral programs, endothelial activation, and downstream thromboinflammation.

## 4 Platelets in Host Defense and Immunity

Platelets, classically indispensable for hemostasis, are equally embedded in the function of innate and adaptive immunity. Their rapid adhesion under shear stress and diverse repertoire of immune receptors position them as sentinels that translate barrier disruption into a coordinated defense termed immunothrombosis, which serves to entrap microbes but, when dysregulated, drives tissue injury through thromboinflammation. In this setting, neutrophils, monocytes, coagulation factors, and platelets form mutually reinforcing loops that favor intravascular containment of pathogens at the cost of potential microvascular occlusion. Platelets catalyze these responses by engaging leukocytes, vWF, fibrin networks, and neutrophil extracellular traps (NETs), thereby linking clot formation to pathogen control [1,[25], [26], [27], [28], [29], [30]].

Platelets regulate cytokine licensing and damage-associated molecular pattern (DAMP)-mediated signaling across innate immune compartments (Figure 2 [23]). They promote interleukin (IL)-1β production by licensing nucleotide-binding oligomerization domain-like receptor family pyrin domain-containing 3 inflammasome expression in innate immune cells, thus influencing systemic inflammation during infection and sterile injury [31]. Beyond, platelets are rich sources of high mobility group box 1, which signals via receptors for advanced glycation end products and TLRs to reinforce protective neutrophil responses in bacterial peritonitis, while simultaneously contributing to thrombosis risk [32]. Platelet serotonin (5-hydroxytryptamine [5-HT]) supports neutrophil recruitment in acute inflammation and, via metabolic processing to 5-hydroxyindoleacetic acid, can act through G protein-coupled receptor 35 to direct granulocyte trafficking from the intestinal-vascular axis to peripheral inflamed sites. In immune complex-driven disease, dysregulated platelet 5-HT can become pathogenic (eg, in shock), illustrating how the same mediator can either promote host defense or amplify collateral damage depending on context [33]. Finally, platelets influence adaptive immunity that reinforces their role as an interface between coagulation and host defense. Platelets can traffic complement-opsonized, blood-borne bacteria to antigen-presenting cells via GPIb and complement C3 [34]. Such functions reinforce memory programming but can also impose brakes during disease. For example, in sepsis, platelet major histocompatibility complex class I restrains CD8^+^ T-cell proliferation and antigen-specific responses, revealing an immunomodulatory checkpoint that may preserve host integrity under overwhelming inflammatory stress [[35], [36], [37], [38]]. Concordantly, in pneumonia-derived gram-negative sepsis, thrombocytopenia worsens bacterial dissemination and enhances systemic cytokine release. Mechanistically, platelet-macrophage interactions can suppress macrophage tumor necrosis factor-α/IL-1β production under strong endotoxin/*Escherichia coli* challenge [39]. Together, these data establish platelets as mediators of the defense-damage balance: they initiate immunothrombosis, foster neutrophil recruitment and NETosis, license inflammasome/IL-1β outputs, and deliver DAMPs and 5HT, while in parallel limiting macrophage and T cell activation, which can either resolve infection or propagate immunopathology, depending on timing, compartment, and inflammatory stimulus.

Beyond acting as adhesive platforms, platelets display a distinctive mechano-scavenger program (Figure 1 [14]). Once adherent in inflamed microvessels, single platelets engage αIIbβ3 and actomyosin to migrate and to bundle fibrin(ogen) together with bacteria on their surface [40]. This cell-autonomous motility depends on lamellipodial actin branching and is abrogated by interference with the Arp2/3 complex or myosin IIa activity. In the pulmonary microvasculature, haptotactic platelets reposition along fibrinogen-exposed inflamed endothelium to patrol and capture microbes efficiently and thereby contribute to effective containment in the lung and limit hematogenous spread [2]. This bundling shapes downstream innate effector functions: when neutrophils encounter platelet-bacteria bundles, they decelerate, phagocytose efficiently, mobilize intracellular Ca^2+^ levels, and undergo NETosis. Importantly, disabling platelet migration increases bacterial spread in a model of bacterial pneumonia. On the other hand, in severe bacteremia, platelet migration and bundling are associated with hepatic neutrophil aggregation and NETosis, thereby worsening organ injury and aggravating mortality. Thus, bundling can potentiate professional phagocyte responses and immunothrombosis that can worsen immunopathology [1,40].

Collectively, the data support a model in which platelets act as dynamic regulators of vascular immunity, integrating coagulation-derived signals into spatially and temporally coordinated immune responses. Bundling and granule secretion constitute complementary effector mechanisms: the former enhances neutrophil engagement and NETosis, while the latter mediates direct antimicrobial activity. In parallel, inflammasome licensing, DAMP and 5-HT release, and antigen presentation via major histocompatibility complex I signaling collectively modulate the magnitude and character of both innate and adaptive immune responses. Understanding when and where these axes are protective versus pathogenic is central to therapeutic strategies that modulate rather than suppress platelet function in infection and inflammation.

### 4.1 Arp2/3 complex-dependent migration and haptotaxis as a novel platelet effector function

The concept of haptotaxis, defined as directional cell migration guided by bound substrate rather than soluble gradients, was introduced by Carter [41] nearly 6 decades ago, who observed cells moving up gradients of adhesiveness. In the platelet field, direct *in vivo* and *ex vivo* evidence has now established that platelets perform Arp2/3 complex-dependent haptotaxis along matrix-bound ligands such as fibrinogen (Figure 1 [14]). Directional platelet motility requires αIIbβ3 integrin ligation and an appropriate adhesive microenvironment [40,42].

The Arp2/3 complex has emerged as a key organizer of inflammatory platelet migration rather than of classical hemostasis. In models of LPS-induced ALI and C-C motif chemokine ligand 2-driven inflammation of the cremasteric microcirculation, platelets initially adhere as single cells to the inflamed microvessels, dynamically extend lamellipodia, and polarize into a half-moon morphology. This allows directed haptotactic migration toward fibrinogen-enriched “hotspots,” most prominently at endothelial junctions and pericyte-deficient wall regions, thereby repositioning themselves to seal microbreaches [2]. Mechanistically, pharmacologic inhibition or genetic ablation of the Arp2/3 complex abolishes lamellipodial dynamics and haptotaxis and converts platelets into filopodia-rich cells with increased nonmotile adherence [2]. In migrating cells, the Arp2/3 complex generates branched actin networks that support lamellipodial protrusion and directionality. Foundational work in fibroblasts showed that the Arp2/3 complex is dispensable for chemotaxis to soluble cues yet essential for haptotaxis and for responding to gradient changes in extracellular matrix concentration [43,44]. In platelets, the Arp2/3 complex concentrates at the leading edge, and acute inhibition with the small-molecule inhibitor CK-666 selectively disrupts lamellipodial cycling and directional migration on fibrinogen gradients [2,45]. Upstream, Rac1 is essential for lamellipodia formation and for aggregate stability under shear, linking Rho-family GTPases to Arp2/3 complex-driven protrusion [42]. Earlier work had already revealed that platelets are autonomously motile: they spread, polarize, and migrate over adhesive plasma proteins under shear. This motility enables platelets to gather and bundle microbes on fibrin-coated surfaces and also migrate in vivo [40].

In sum, these studies converge on a key principle: platelets utilize Arp2/3 complex-mediated lamellipodia to detect and migrate along immobilized matrix gradients, thereby repositioning precisely to sites of vascular injury and pathogen engagement. This represents a newly recognized effector function within the broader framework of platelet immune surveillance [2,40,46].

## 5 Platelets and Inflammatory Bleeding

Classical (traumatic) hemostasis depends on the extensive recruitment, activation, and aggregation of platelets within a fibrin scaffold to generate a stable hemostatic plug. In contrast, during inflammation, the vascular barrier is disrupted at sites of leukocyte diapedesis, where the formation of an occlusive thrombus would be detrimental. Here, single “immune-responsive” platelets patrol, migrate to, and seal microlesions without building classical aggregates, using a distinct repertoire of effector functions and receptors [14,[47], [48], [49], [50]] (Figure 1 [14]).

Blocking neutrophil diapedesis prevents inflammatory hemorrhage during thrombocytopenia, underscoring the critical role of platelet-leukocyte interactions in vascular barrier disruption [51]. Complementing this, in inflamed microvessels, platelets use Arp2/3 complex-dependent lamellipodia to follow along 2-dimensional fibrinogen gradients, repositioning precisely to microlesions to prevent bleeding [2]. Collectively, these data establish inflammatory hemostasis as a single-cell, nonocclusive mode of vascular protection that is mechanistically distinct from traumatic hemostasis [14,52].

One aspect that remains incompletely understood is how platelets seal microinjuries upon engagement. A proposed mechanism involves direct physical coverage of the damaged area through the extension of platelet lamellipodia. Notably, Cyfip1-deficient platelets, which lack the WAVE regulatory complex, retain migratory capacity but are unable to form large lamellipodia. Despite this deficiency, Cyfip1 loss does not exacerbate inflammatory bleeding, indicating that surface enlargement and direct physical coverage are not essential for platelet-mediated inflammatory hemostasis [2,20]. Recent work suggests a different mechanism: local engagement of plasmatic coagulation on platelet surfaces. In an LPS-induced ALI model, either thrombocytopenia or pharmacologic blockade of factor Xa (rivaroxaban/enoxaparin) or factor IIa (argatroban) markedly exacerbated alveolar hemorrhage, while neutrophil recruitment remained unchanged. Histology and live imaging revealed fibrinogen deposition on phosphatidylserine (PS)-positive platelets at the luminal side, and platelet depletion markedly reduced this fibrinogen signal [53]. Together with the sentinel function described above, these findings suggest that platelets actively recruit and localize coagulation to endothelial microdefects, thereby limiting hemorrhage without inducing vascular occlusion [14].

## 6 Procoagulant Platelets as Immune Effectors

Procoagulant activation (PA) marks a terminal effector program in a subset of platelets that is mechanistically and functionally distinct from classical aggregatory activation. Upon cooperative strong stimulation, platelets undergo a transformation characterized by supramaximal cytosolic Ca^2+^ level elevations, mitochondrial depolarization mediated by the cyclophilin-D-dependent mitochondrial permeability transition pore, cell swelling/ballooning, PS externalization via the Ca^2+^-activated scramblase transmembrane protein 16F (TMEM16F; *anoctamin-6*), and localized accumulation of fibrinogen on the platelet surface [[54], [55], [56], [57]]. This procoagulant transformation creates a competent surface for prothrombinase assembly and thrombin generation, thereby coupling sustained platelet activation to local coagulation (Figure 2 [23]). Experimental in vitro studies using strong dual stimulation with GPVI-agonists and thrombin indicate that ∼30% to 60% of platelets convert to a PS-exposing, procoagulant phenotype [57]. Under physiological conditions, procoagulant platelets are generated locally at sites of vascular injury and remain rare in the circulation of healthy individuals (< 1%). By contrast, pathologic thromboinflammatory states are associated with both elevated circulating levels and exaggerated agonist-induced formation of procoagulant platelets. Increased frequencies of PS-exposing platelets have been reported in patients with transient ischemic attack and stroke, cancer-associated thrombosis, severe COVID-19, and venous thromboembolism [[57], [58], [59], [60]]. In some settings, procoagulant platelets can comprise more than 10% of the total circulating platelet pool. Thus, PA platelets represent a numerically restricted but functionally specialized subset within the activated platelet pool.

Procoagulant platelets arise when experiencing strong signaling downstream of immunoreceptor tyrosine-based activation motif receptors, together with integrin outside-in signaling. In classical models, this has been best characterized for GPVI engagement by collagen. However, polymerized fibrin and fibrinogen also serve as physiologic GPVI ligands and cooperate with GPIIbIIIa to promote PS exposure, thrombin generation, and clot stabilization [15,16,61]. These observations indicate that procoagulant platelets are not confined to settings with collagen exposure but can be generated more broadly in fibrin-rich thromboinflammatory environments. VWF-GPIb interactions are crucial for platelet docking to inflamed endothelium, primarily acting as adhesion and priming. Additionally, inflammatory GPIb ligands such as S100A8/A9 can directly induce PS-positive, procoagulant platelets and foster fibrin-dependent immunothrombosis in diseases such as COVID-19 [14,62,63].

Mechanistically, live imaging of inflamed microvessels reveals that the interaction of individual platelets with subendothelial matrix components, such as collagen, induces platelet ballooning and PS exposure [53]. Morphologically, procoagulant platelets localize to vessel surfaces where they colocalize with fibrinogen [64]. The same phenotype also occurs in stenosis-induced venous thrombosis [60]. At the receptor/signaling level, platelet PA integrates GPVI-Syk-PLCγ2 signaling with integrin αIIbβ3 (GPIIbIIIa) outside-in signals. Engagement of GPVI on collagen triggers sustained Ca^2+^ signaling and PS exposure under flow conditions and in vivo [65,66]. Moreover, the GPVI–PLCγ2 signaling axis is essential for collagen-induced PS exposure and fibrin formation in both venules and arterioles [67]. GPIIbIIIa outside-in signaling, initiated after ligand binding and integrin clustering, recruits Src-family kinases and Syk that set the cytoskeletal and membrane context for PA [68,69]. In inflammatory settings, GPIIbIIIa-Gα13-c-Src signaling acts together with GPVI to raise Ca^2+^ levels above the PA threshold. Combined GPIIbIIIa and GPVI interference reduces PA in vivo [53]. Finally, water and proton homeostasis modulate the ballooning/PS pathway. Aquaporin-1-mediated water influx facilitates platelet ballooning, while carbonic anhydrase activity regulates intracellular pH buffering that permissively supports PS exposure [70].

In ALI and mesenteric inflammation, platelets patrol the endothelium, sense exposed subendothelial collagen, arrest, and transition to a procoagulant phenotype, thereby inducing local fibrin formation specifically at endothelial breaches generated by neutrophil transmigration (Figure 1 [14]). Genetic ablation of CypD or TMEM16F selectively aggravates inflammatory bleeding (alveolar hemorrhage) without altering neutrophil recruitment [53]. When platelet PA is amplified or disseminated, it shifts from local protective effects to systemic risk. In human pulmonary emboli retrieved by thrombectomy, procoagulant platelets are readily detectable within the clot. In murine inferior vena cava stenosis, the circulating burden of PS^+^ platelets correlates with thrombus weight, and intravital imaging reveals procoagulant platelet-rich niches within developing venous thrombi [60].

Taken together, these findings establish procoagulant platelets as a distinct immune effector function. They sense vascular damage through collagen and flow cues, couple innate signaling to local coagulation, and safeguard vessel integrity during inflammation. However, when dysregulated or systemically amplified, this same program drives (venous) thrombosis.

## 7 Functional Specialization of Platelets Across Their Lifespan

Platelets are not a uniform population: their effector repertoire evolves over their short life in the circulation. Converging evidence from integrated in vivo labeling, functional assays, and proteomics demonstrates an age-linked division of labor, with newly released, reticulated platelets (RPs) optimized for hemostasis and progressively aged platelets shifting toward immune-effector functions [[71], [72], [73], [74], [75]].

Using pulse-labeling in mice, platelets can be tracked in vivo as 0- to 12-hour (“young”) or 96- to 108-hour (“aged”) populations, enabling quantitative assessment of their phenotype and function across hemostatic and inflammatory contexts. Young platelets display enhanced responses to classical agonists like collagen and ADP, superior adhesion and spreading, and greater force generation during clot retraction [71]. Similarly, RPs exhibit heightened reactivity and initiate aggregation even under antiplatelet therapy, further supporting their specialized hemostatic function [73]. In contrast, aged platelets exhibited increased baseline PS exposure with enhanced predisposition for procoagulant transformation, augmented platelet-leukocyte aggregate formation, and effective bacterial binding as well as killing. Phenotypically, aged platelets showed increased expression of CD40 ligand (CD40L), intercellular adhesion molecule 2, and C-type lectin-like receptor 2, known receptors important for platelet immune function. In addition, proteomics revealed increased immunoglobulin and complement content in aged platelets. Correspondingly, in vivo, aged platelets were preferentially recruited to the inflamed lung and enriched in bronchoalveolar lavage following LPS-induced ALI, whereas young platelets predominated in thrombus formation under flow [71].

The number of young RPs released into the bloodstream is dynamically tuned by inflammatory cues. Neutrophil “plucking” of proplatelet buds through C-X-C motif chemokine receptor type 4-C-X-C motif chemokine ligand 12 signaling accelerates the final stages of thrombopoiesis, enhancing the release of immature platelets during thromboinflammation and thereby increasing the prothrombotic, metabolically active platelet fraction [76]. In parallel, nonbone marrow platelet production sites produce context-specific platelet phenotypes [77]. The lung contributes substantially to platelet production through resident megakaryocytes that respond dynamically to pneumonia [78]. During sepsis, extramedullary megakaryopoiesis in the spleen produces CD40L-high platelets that potentiate neutrophil effector functions and promote NET formation, exemplifying tissue-of-origin imprinting that can override platelet age-related programming [79].

Platelet lifespan is determined by B-cell lymphoma-extra large protein levels, which restrain BAK/BAX-mediated intrinsic apoptosis. Genetic deletion of BAK and BAX in platelets doubles their lifespan and increases platelet counts but results in an accumulation of aged platelets with impaired hemostatic function. These platelets exhibit prolonged bleeding times, unstable thrombi, reduced agonist-induced integrin activation and aggregation, and defective granule release [80]. At the same time, the formation of platelet-leukocyte aggregates and procoagulant transformation is enhanced, leading to increased thromboinflammation in a model of ALI.

These findings highlight that platelet heterogeneity originates from megakaryocyte diversity, distinct sites of biogenesis (bone marrow, lung, and spleen), and differences in circulatory age. Within this conceptual model, platelet “cell age” emerges as a principal axis shaping hemostatic and immunothrombotic effector functions across the circulating platelet pool [72]. While the observed functional and phenotypic adaptation points toward an active process, the underlying mechanisms triggering an “aged inflammatory” platelet phenotype remain to be explored. Beyond ontogeny and age-dependent differences, platelet heterogeneity also emerges from stimulus-specific sensing of exogenous pathogen-associated molecular patterns and endogenous DAMPs. Platelets express multiple pattern-recognition receptors, including TLRs (eg, TLR2, TLR4, and TLR7/9) and C-type lectin-like receptors such as C-X-C motif chemokine ligand 2, which allow them to discriminate between infectious and sterile inflammatory cues [25,26].

## 8 Further Directions and Therapeutic Outlook

**Figure 3:**
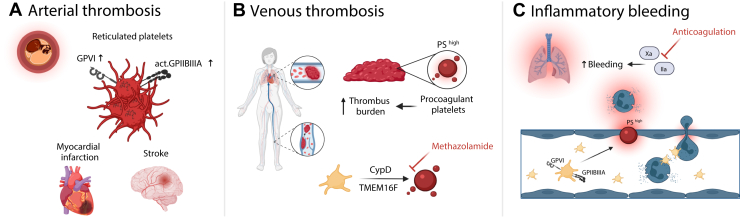
Platelet effector functions in different disease models (A) Arterial thrombosis. Schematic of reticulated (immature) platelets highlighting their larger size and heightened reactivity. These young platelets display exaggerated GPVI signaling and enhanced integrin αIIbβ3 activation, promoting rapid aggregation and thrombus growth. Clinically, elevated reticulated platelet counts are observed in acute myocardial infarction and ischemic stroke. (B) Venous thrombosis (DVT/PE). Within venous thrombi, procoagulant platelets (ballooned, phosphatidylserine-exposing platelets) accumulate and track with thrombus burden. Genetic disruption of cyclophilin D (CypD) or the scramblase TMEM16F limits procoagulant conversion and mitigates thrombosis. Pharmacologic inhibition of carbonic anhydrase (eg, methazolamide) similarly reduces the procoagulant platelet response and venous clot formation. (C) Inflammatory bleeding (lung model). During neutrophil-driven endothelial injury, GPVI costimulation with αIIbβ3 converts a subset of platelets to a procoagulant phenotype that seals microvascular breaches and limits hemorrhage. Therapeutic anticoagulation, illustrated as factor Xa or IIa inhibition, diminishes local thrombin generation by these platelets and exacerbates bleeding in inflamed tissue (created by biorender.com). DVT, deep vein thrombosis; GP, glycoprotein; PE, pulmonary embolism.

Platelets have emerged as sentinels that both initiate innate immune programs and preserve vascular integrity during inflammation. Intravital and systems-immunology studies now place platelets at the earliest checkpoints of inflammation, where they modulate neutrophil behavior (adhesion mode, motility state, and effector output) and couple these programs to vascular barrier protection. Research of recent years outlines how core platelet effector functions such as adhesion, migration/haptotaxis, and PA are deployed according to tissue microenvironmental cues and endothelial priming [1,72,81]. Moving forward, 2 complementary priorities emerge: first, the mechanistic dissection of context-specific platelet effector functions that regulate leukocyte behavior and vascular barrier integrity; and second, the translation of these insights into therapeutic strategies that mitigate thromboinflammation while preserving inflammatory hemostasis [56] (Figure 3).

PA of individual immune-responsive platelets is essential for preventing inflammatory microbleeding. However, these PA programs are of minor importance for traumatic (“bulk”) hemostasis [14]. This distinction raises the possibility that selectively attenuating PA in specific contexts could mitigate thromboinflammation while minimizing the bleeding risk associated with conventional global antiplatelet therapy such as COX- and P2Y12-inhibitors [14]. Pharmacologic interference with PA, for example, through the carbonic anhydrase inhibition by methazolamide, reduces circulating PS^+^ platelets, decreases deep vein thrombosis incidence and thrombus burden following inferior vena cava stenosis, while preserving trauma-associated hemostasis [60]. More promising strategies may instead focus on disease-restricted upstream drivers that selectively amplify PA in thromboinflammatory settings, such as S100A8/A9-GPIbα signaling in severe infection [62]. These findings support PA attenuation as a therapeutic strategy with a favorable protection-to-bleeding profile.

Targeting the formation of PNAs through platelet depletion, PSGL-1 deficiency, or acute PSGL-1 blockade selectively impairs neutrophil crawling and attenuates NET release. This can limit pulmonary edema in transfusion-related ALI and decrease vascular injury in models of endotoxemia and stroke [10].

Recent advances in transfusion medicine recognize that platelet products are inherently heterogeneous. Younger, reticulated (immature) platelets more effectively support plug formation, clot retraction, and platelet recruitment under shear conditions, whereas aged platelet populations exhibit immunomodulatory and procoagulant phenotypes that could exacerbate tissue injury in vulnerable hosts [71]. Some clinicians advocate for the routine reporting and selection of platelet products based on immature platelet fraction (%) and related functional surrogates to support indication-aligned transfusion strategies, thereby reinforcing the principles of precision medicine [72].

The field is transitioning from broad antiplatelet approaches toward precise modulation of platelet immune effector functions. A practical approach favors modulation of PA-specific pathways over global platelet activation, interventions at platelet-neutrophil interfaces, and age-aware transfusion practices that align product biology with clinical indication. Done well, this approach separates vascular protection from pathogen control and antithromboinflammatory therapies from inflammatory bleeding risk.

### 8.1 International Society on Thrombosis and Haemostasis 2025 congress report

At the 2025 International Society on Thrombosis and Haemostasis conference, several studies advanced a coherent view of platelets as immune effectors whose inflammatory behavior was set upstream by megakaryocyte programming, refined by platelet-intrinsic checkpoints, and executed through cross-talk with leukocytes across infection, fibrosis, vascular, and renal disease. Collectively, this work emphasized mechanisms that shaped procoagulant programming and thromboinflammatory outcomes while suggesting potential points for intervention.

### 8.2 Intracellular platelet signaling and programming

Multiple reports placed megakaryocyte programming and platelet-intrinsic checkpoints at the center of inflammatory and procoagulant control. Protein arginine methyltransferase 1 regulates immune megakaryopoiesis; a protein arginine methyltransferase 1-Dusp4 axis links megakaryocyte fate to platelet inflammatory potential and yields cytokine-rich, hyperreactive platelets [82]. During viral infection, platelets engage retinoic acid-inducible gene I (*RIG-I*) signaling to counterbalance thrombosis via a granule-independent S100A10 pathway, indicating that intrinsic antiviral programs could be antithrombotic in vivo [83]. In endotoxemia models, proline-rich tyrosine kinase 2 (Pyk2) is required for amplified platelet activation and platelet-neutrophil coupling; its loss attenuated microvascular thrombosis, suggesting Pyk2 as a candidate node early in thromboinflammation [84].

Downstream of receptors, control of the mitochondrial permeability transition pore appeared to separate pathologic procoagulant programming from primary hemostasis. Two small-molecule mitochondrial permeability transition pore inhibitors reduced PS exposure and delayed fibrin generation while preserving aggregation, supporting the concept of antithrombotics that targeted procoagulant, rather than aggregatory, platelets [85]. Cytoskeletal scaffolding also matters: filamin A enabled thrombin-evoked Src/p21-activated kinase phosphorylation and p21-activated kinase 1 membrane translocation. Platelet filamin A loss reduced eosinophil recruitment in a papain asthma model, linking platelet signaling to allergic airway inflammation [86]. Transmembrane disulfide isomerase Thioredoxin-related transmembrane protein 1 (TMX1) provides a brake on the procoagulant response: genetic loss of TMX1 increased PS exposure and fibrin formation, with mechanistic data indicating that TMX1 oxidizes TMEM16F cysteines and suppresses αIIbβ3 outside-in signaling, thereby reducing thrombin generation and maintaining vascular quiescence [87]. Pattern-recognition inputs also tune procoagulant programming: TLR2 agonists increase PS exposure, fibrin generation, and platelet-neutrophil aggregation, whereas CD36 blockade selectively attenuates TLR2-driven, but not protease-activated receptor 4-driven, responses, consistent with CD36 acting as a costimulatory amplifier of TLR-evoked procoagulant signaling [88]. Finally, age and sex appear to imprint basal risk at the progenitor level: in males, increased scramblase and reduced flippase expression bias platelets toward PS externalization, motivating consideration of sex-aware risk stratification along the scramblase-flippase axis [89].

### 8.3 Intercellular platelet cross-talk in thromboinflammation

Platelet-neutrophil interplay is a common driver of thrombus composition and organ injury across sepsis, venous thrombosis, autoimmunity, and cytokine storm. In sepsis, PSGL-1^high^ neutrophils transport and deposit procoagulant platelets at the thrombus periphery, producing dense, fibrin-rich architecture; targeting procoagulant platelets with lactadherin, depleting neutrophils, or blocking PSGL-1 reduced thrombus size and normalized architecture [90]. In deep vein thrombosis, neutrophil CD14 promotes NETosis, S100A8/A9 release, and accumulation of procoagulant platelets within thrombi. Neutrophil-specific CD14 knockdown decreases thrombus burden and accelerates resolution, highlighting CD14 as a neutrophil-focused antithromboinflammatory strategy [91]. In systemic sclerosis models, platelet activation via GPVI precedes neutrophil activation and peptidylarginine deiminase-4-dependent NET release. Platelet depletion or GPVI deficiency reduces platelet/neutrophil activation and is protective in 2 murine models, defining a GPVI-platelet-neutrophil-NET axis that links inflammation to fibrosis [92].

### 8.4 Platelets in infection, fibrosis, and chronic disease

Across parasitic infection, pulmonary fibrosis, diabetic kidney disease, and pulmonary hypertension, platelets act as immune effectors. In malaria, the platelet mammalian target of rapamycin signaling amplifies platelet activation, vascular leakage, and monocyte recruitment, worsening cerebral disease. Platelet-specific mammalian target of rapamycin deletion improves survival, reduces brain vascular permeability, and preserves neurologic function without altering parasitemia or thrombocytopenia, indicating a platelet-intrinsic contribution to neuropathogenesis [93]. In chronic thromboembolic pulmonary hypertension, platelet proteomics identifies 179 dysregulated proteins with upregulation of NADPH oxidase 2, peptidylarginine deiminase-4, integrin subunit β 2, and high mobility group box 1, while increased P-selectin and myeloperoxidase-DNA associate with clinical indicators, nominating platelet signatures as candidate biomarkers and potential targets [94]. In idiopathic pulmonary fibrosis and bleomycin-treated mice, platelets accumulated within fibrotic regions. Activation of the GPVI-PF4 axis promotes fibroblast differentiation and collagen synthesis, whereas GPVI or PF4 deficiency is protective in bleomycin-induced fibrosis, implicating this pathway in fibrogenesis [95]. Hyperglycemia drives platelet activation and platelet-neutrophil aggregation, leading to NET formation and endothelial dysfunction. In diabetic kidney disease, aspirin or PSGL blockade reduced albuminuria and restored glomerular markers, linking platelet-NET cross-talk to renal injury [96]. During interferon γ-driven cytokine storm, platelets become degranulated, procoagulant, and aggregatory, forming lung and liver microthrombi that contribute to tissue damage; platelet depletion mitigates organ injury without altering weight loss or cytopenia [97].

### 8.5 Translational and clinical perspectives

In primary antiphospholipid syndrome, low-grade inflammation amplifies antidomain 1 of β2-glycoprotein immunoglobulin G-driven platelet activation: IL-6 augments P-selectin and activates GPIIb/IIIa. Aspirin and P2Y12 inhibition suppress activation, whereas hydroxychloroquine affects adhesion but does not blunt tissue factor expression, arguing against hydroxychloroquine monotherapy for thromboinflammatory control [98]. Amyloid-β (1–42) drives platelet activation, enhancing aggregation, degranulation, αIIbβ3 activation, and procoagulant programming. Procoagulant platelets colocalize with amyloid fibrils, and Ca^+^ chelation or FXIII-A inhibition reduces fibril formation and procoagulant platelets, pointing to platelet-amyloid interactions as a potential target in Alzheimer’s disease [99]. Finally, platelet-derived extracellular vesicles from platelet-rich plasma restore retinal layer thickness and reduce edema and degeneration in a streptozotocin model of diabetic retinopathy, supporting their therapeutic potential [100].

### 8.6 Platelet function in transfusion medicine

In healthy volunteers, autologous platelet transfusion does not alter the plasma proteome at baseline or during inflammation; in thrombocytopenic hemato-oncology patients, allogeneic transfusion increases CXCL7, but this is not associated with bleeding severity or time to major bleed [101]. Donor sex emerged as a determinant of storage stability in lipidomic analyses: male cold-stored platelets exhibit reduced aggregation and impaired viscoelastic function over time, whereas female donor function is associated with enrichment of hydrolyzed phospholipids [102]. Baseline metrics such as residual red cell content and rotational thromboelastometry parameters, but not platelet count, glucose, or pH, predict preserved function after 21 days at 4 °C, suggesting a path toward precision donor selection [103]. Additive solutions also influenced clot stability: phosphate-containing solutions impair fibrin network formation and accelerate fibrinolysis, whereas Composol maintains stable, cross-linked fibrin structures [104]. A scalable microfluidic high-shear assay shows that both room-temperature and cold-stored platelets restore clot formation and stability in trauma samples, aligning with rotational thromboelastometry outcomes and enabling real-time evaluation as a complement to conventional testing [105].

## 9 Conclusion

Across diverse models, sepsis and venous thrombosis, systemic sclerosis, malaria, pulmonary fibrosis, diabetic kidney disease, pulmonary hypertension, and retinal injury, these abstracts depict platelets as immune effectors whose inflammatory programming is set by megakaryocyte imprinting, tuned by platelet-intrinsic sensing and signaling, and amplified through platelet-neutrophil cross-talk. From a transfusion standpoint, donor factors, storage chemistry, and functional assessment together could improve product performance.
